# DTI-HeNE: a novel method for drug-target interaction prediction based on heterogeneous network embedding

**DOI:** 10.1186/s12859-021-04327-w

**Published:** 2021-09-03

**Authors:** Yang Yue, Shan He

**Affiliations:** 1 College of Information and Electrical Engineering, China Agricultural University, Beijing, 100083 China; 2 Centre for Computational Biology, School of Computer Science, The University of Birmingham, Edgbaston, Birmingham, B15 2TT UK

**Keywords:** Drug-target interaction prediction, Heterogeneous network embedding, Graph mining, Feature fusion

## Abstract

**Background:**

Prediction of the drug-target interaction (DTI) is a critical step in the drug repurposing process, which can effectively reduce the following workload for experimental verification of potential drugs’ properties. In recent studies, many machine-learning-based methods have been proposed to discover unknown interactions between drugs and protein targets. A recent trend is to use graph-based machine learning, e.g., graph embedding to extract features from drug-target networks and then predict new drug-target interactions. However, most of the graph embedding methods are not specifically designed for DTI predictions; thus, it is difficult for these methods to fully utilize the heterogeneous information of drugs and targets (e.g., the respective vertex features of drugs and targets and path-based interactive features between drugs and targets).

**Results:**

We propose a DTI prediction method DTI-HeNE (DTI based on Heterogeneous Network Embedding), which is specifically designed to cope with the bipartite DTI relations for generating high-quality embeddings of drug-target pairs. This method splits a heterogeneous DTI network into a bipartite DTI network, multiple drug homogeneous networks and target homogeneous networks, and extracts features from these sub-networks separately to better utilize the characteristics of bipartite DTI relations as well as the auxiliary similarity information related to drugs and targets. The features extracted from each sub-network are integrated using pathway information between these sub-networks to acquire new features, i.e., embedding vectors of drug-target pairs. Finally, these features are fed into a random forest (RF) model to predict novel DTIs.

**Conclusions:**

Our experimental results show that, the proposed DTI network embedding method can learn higher-quality features of heterogeneous drug-target interaction networks for novel DTIs discovery.

## Background

Drug repurposing or repositioning refers to deploying old drugs for new purposes, which holds great promise in the future. That is because developing a new drug is costly and time-consuming [1]. By contrast, drug repurposing, i.e., finding the new use of existing drugs approved by the Food and Drug Administration (FDA) could save time and experimental funds for clinical trials. DTIs prediction based on computational techniques plays an important role in drug repurposing because it requires lower cost and less time, compared with biochemical experimental methods [2–4]. With an increasing number of public databases [5], different computational strategies can be more effectively applied for the DTIs prediction. There are two varieties of traditional computational methods: the ligand-based method [6] and the structure-based or docking-based method [7], which can provide relatively accurate DTI predictions. However, the former one has the limitation on predictive performance when few binding ligands are provided for a certain target, while the latter will not be feasible when the three-dimensional (3D) structure of the target is not available [2].

In recent years, machine-learning-based methods have been widely used for the DTIs prediction because they can search more potential targets of existing drugs in the DTIs space. The main assumption of most of these methods is that similar drugs may share similar targets [8]. Based on this assumption, kernel-based methods have been proposed, which essentially map various drug-drug and target-target similarity matrices (i.e., kernels) to DTI labels [9, 10].

A recent trend is the graph-based methods, compared with kernel-based methods, they can better describe interactive relations between drugs and targets by vertices and edges. The methods extract topological features from drug-target interaction networks and process these features for DTI predictions [11]. However, many existing methods cannot consider the distinctive characteristics carried by different types of entities and complex relations between these entities. Heterogeneous information networks are powerful tools to model the semantic information of such complex data by varieties of vertices and edges [12]. It is natural to use heterogeneous networks to represent the characteristics of drug and target vertices as well as diverse relations between drugs and targets. After constructing heterogeneous DTI networks, we need to use network embedding algorithms to extract the features, i.e., low-dimensional vector representations of networks, for downstream machine learning tasks, e.g., link predictions [13, 14].

However, while many homogeneous network embedding algorithms exist and have been applied to DTI predictions, heterogeneous network embedding remains a challenging task due to the various vertex types and the diversity of relations between vertices. Recently, Chen et al. [15] proposed an idea to cope with the heterogeneous network embedding: a heterogeneous network can be decomposed into several sub-networks, and each of them is processed separately. Similarly, a heterogeneous DTI network can be divided into a bipartite DTI network and other auxiliary networks which contain similarity information between the same kind of nodes. Luo et al. [2] proposed an approach named DTINet, which could learn embeddings by the network diffusion algorithm and inductive matrix completion strategy. Based on a heterogeneous network, Thafar MA et al. [16] utilized node2vec [17], graph mining techniques, and drug and target similarities generated by heuristic algorithms for DTI predictions. Peng et al. [18] introduced a random walk with restart (RWR) model, a denoising autoencoder (DAE), and a Convolutional Neural Network (CNN)-based model to extract low-dimensional vectors from heterogeneous networks, and they also used end-to-end graph convolutional networks (GCN) to do the similar work [19].

Although the methods mentioned above achieved promising results, there are still some issues. More specifically, current methods do not explicitly consider the bipartite nature of the drug-target interactions (containing all known DTIs) in a heterogeneous DTI network. Instead, these bipartite drug-target interactions are treated equally with other auxiliary information such as drug-drug and target-target similarity information. Such an indiscriminate treatment of heterogeneous relationships might lead to the suboptimal set of features and will ultimately affect the accuracy of the DTIs prediction.

To address this issue, we propose a novel heterogeneous network embedding method called DTI-HeNE that specially considers the bipartite drug-target relations. Similar to Chen et al. [15], we first decompose a heterogeneous DTI network into a bipartite DTI network and homogeneous drug-drug and target-target similarity networks. The proposed method is a multi-staged embedding method with good interpretability which then employs Bipartite Network Embedding (BiNE) [20] to specifically learn the DTI embeddings from the bipartite DTI network. Next, a path-based method is developed to combine the bipartite DTI embeddings with the homogeneous networks according to the topological information of pathways between sub-networks for creating new embedding representations of all drug-target pairs. Finally, we acquire novel DTIs by running a random forest (RF) model to learn these integrated representations.

## Methods

### Problem formulation

In our study, the DTIs prediction can be formulated as a transductive-learning binary link-prediction task (i.e., discovering novel DTIs within the DTIs space consisted of fixed drugs and targets in the given dataset, that is, the involved entities do not need to be extended) based on a heterogeneous network, which is divided into a bipartite DTI network as well as drug and target homogeneous networks. More specifically, let ${G}_{b}=(\mathrm{D},\mathrm{ T},\mathrm{ E})$ be a bipartite DTI network, where $\mathrm{D}=\{{d}^{1}, {d}^{2},\dots ,{d}^{m}\}$ (*m* refers to the number of drugs in the dataset) and $\mathrm{T}=\{{t}^{1}, {t}^{2},\dots , {t}^{n}\}$ (*n* represents the amount of targets in the dataset) denote the set of drug and target protein nodes, respectively. $\mathrm{E}\subset \mathrm{D}\times \mathrm{T}$ defines known edges (interactions) between drugs and targets, and all known edges correspond to the weight of 1. Meanwhile, the homogeneous drug and target networks are defined as the $m\times m$ matrix (${G}_{d}$) and the $n\times n$ matrix (${G}_{t}$), respectively, in which every element indicates the degree of similarity between two drugs or two targets. The higher the value of one element, the higher the similarity between two corresponding entities. In addition, there is a $m\times n$ matrix (Y) storing binary DTI predictions, if ${y}^{ij}=1$, it indicates that the ${d}^{i}-{t}^{j}$ pair is predicted to have a potential interaction, if not, then ${y}^{ij}=0$.

Furthermore, it is precisely because of the definition of our prediction task (i.e., the involved nodes are fixed) that the transductive-learning-like method can be utilized. Another contributing reason is that directly setting the weight of unknown interactions to 0 may not produce a satisfactory performance on datasets with a highly imbalanced ratio between known and unknown samples (e.g., DTI datasets) [21]. The transductive learning allows methods to have observed all the data beforehand, including training and test datasets, and potentially exploit structure information in their distribution [22] (so that it can better use additional information of unknown samples in the face of datasets with sparse known interactions). Compared with the inductive learning that learns a general inference to a task based on the information of a dataset, transductive learning is less ambitious and finds a specific solution that is optimal only for the current dataset (i.e., acquiring the best performance under the fixed drugs and targets in the dataset in our case study) [23, 24]; and the transductive setup has already been mentioned by some DTI prediction approaches [25].

### Workflow

**Fig. 1:**
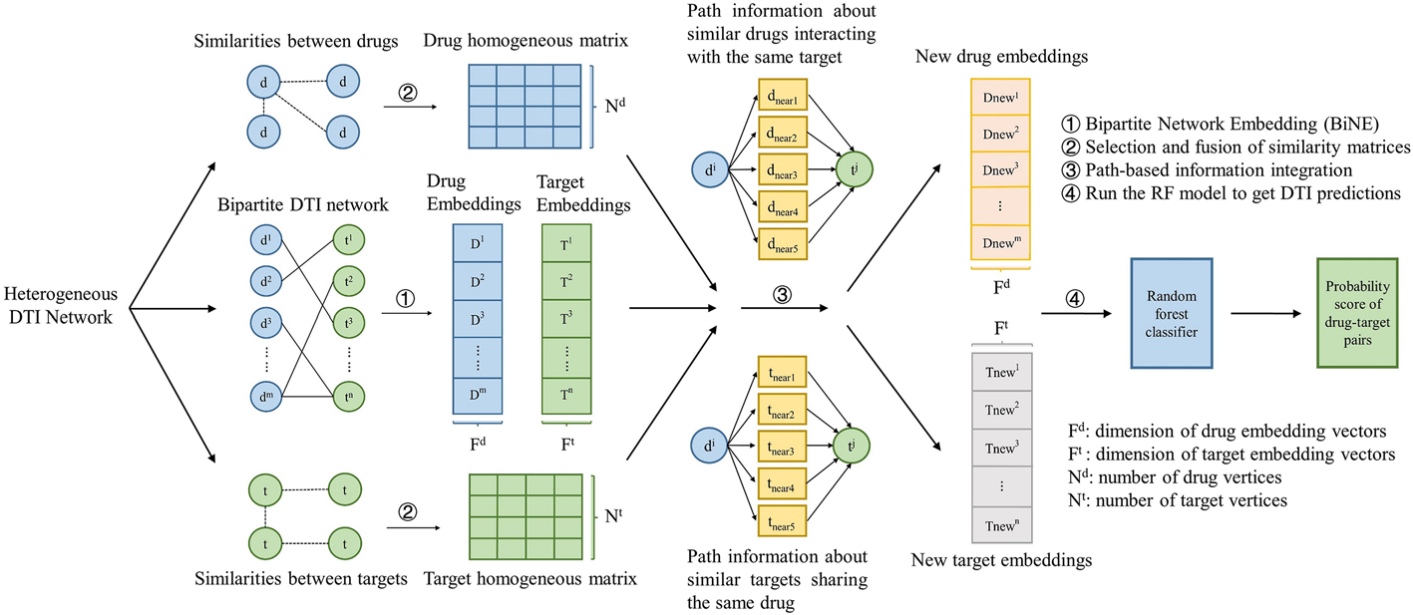
The flowchart of our method. The method integrates three varieties of networks to acquire embeddings of drug-target pairs. The original representations of drug and target nodes are produced by BiNE, and then these representations are augmented using the drug and target homogeneous matrices as well as path-based topological features for predicting DTIs

Figure 1 presents the four main steps of the proposed method in our study:

1. Obtaining drug and target embeddings: a bipartite DTI network is established based on every known ${d}^{i}-{t}^{j}$ pair, and then the BiNE algorithm is performed on the bipartite network to capture the prior high-order similarity information of explicit and implicit transition relationships of all entities in the dataset.
2. Selection and fusion of homogeneous networks: a heuristic algorithm is applied to screen and integrate multiple drug and target homogeneous networks.
3. Path-based information integration: in this step, the path-based heterogeneous information is added as the auxiliary information to generate the embedding of every ${d}^{i}-{t}^{j}$ pair.
4. Novel DTI predictions: a RF classifier is trained to learn the integrated embedding representations for predicting unknown DTIs.

### Learning bipartite DTI embedding

The challenge of learning bipartite network embedding is how to learn the explicit bipartite relationships between different types of vertices (e.g., DTIs) and implicit transition relationships between the same types of vertices (e.g., drugs and targets) simultaneously. BiNE addresses this challenge by using a three-part joint optimization framework and assigns each type of relationships with a dedicated objection function and an adjustable weight, which produces better vertex embeddings. Specifically, the first part of the framework is modeling the explicit relationships. In order to preserve the information of observed edges between two different types of nodes (${u}_{i}$ and ${v}_{j}$), the KL-divergence is chosen to measure the difference between the joint probability $P(i,j)$ between vertices ${u}_{i}$ and ${v}_{j}$ and the joint probability $\widehat{P}(i,j)$ between the embedding vectors of vertices ${u}_{i}$ and ${v}_{j}$ ($\overrightarrow{{u}_{i}}$ and $\overrightarrow{{v}_{j}}$). The objection function can be defined as follows, which aims at minimizing the difference between $P(i,j)$ and $\widehat{P}(i,j)$:

1 $$\mathrm{minimize }{O}^{1}=KL(P||\widehat{P})=\sum_{{e}_{ij}\in E}P(i,j)\mathrm{log}(\frac{P(i,j)}{\widehat{P}(i,j)})$$

**Fig. 2:**
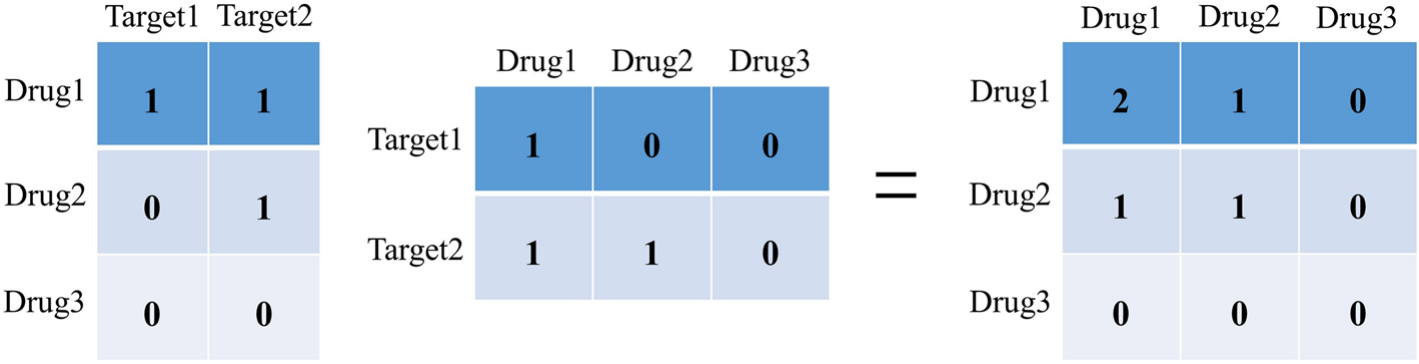
An illustration of the drug homogeneous network generated in BiNE. Assuming that there are only three drugs and two targets in the whole bipartite DTI matrix ${G}_{b}$. When the $3\times 3$ drug homogeneous matrix is made by multiplying ${G}_{b}$ (the $3\times 2$ matrix) by ${G}_{b}^{T}$ (the $2\times 3$ matrix), we can find that, in this $3\times 3$ matrix, the value between Drug1 and Drug2 is 1, while the value between Drug1 and Drug3 is 0, and these values correspond to the DTI relations in ${G}_{b}$. Specifically, Drug1 and Drug2 share one target (Target2), Drug1 and Drug3 do not share any target, correspondingly, the value of the former drug pair in the drug homogeneous matrix is higher than that of the latter one

For the sake of explicitly modeling the unobserved but transitive links (implicit transition relationships) between the same type of nodes (i.e., directly modeling that similar drugs/targets could interact with similar targets/drugs in our case study), firstly, BiNE utilizes an idea named Co-HITS [26] to generate two homogeneous networks (matrices) which contain the 2nd-order proximity between the same type of nodes, and then the nodes having at least one weight greater than 0 are selected in the generated matrices. Then the truncated random walks, which are designed to better capture the frequency distribution of nodes, are performed on these two homogeneous networks consisted of selected nodes respectively, to convert the networks into two corpora of vertex sequences. More specifically, during our DTIs prediction process, there are two different types of homogeneous networks being generated. The first type is obtained in the second step of the workflow shown in Fig. 1, which contains the chemical and physical similarity information of drugs and targets and is more widely used by other DTIs prediction methods [27]. For the second type, it is calculated by Co-HITS mentioned above to model the implicit transition relationships, which has the same size as the first type (i.e., drug homogeneous networks: $m\times m$ matrix, target homogeneous networks: $n\times n$ matrix), and every element (weight) denotes the implicit transition probability between two drugs/targets. That is, given a $m\times n$ bipartite DTI matrix ${G}_{b}$, the drug homogeneous network can be represented by a $m\times m$ matrix ${G}_{b}{G}_{b}^{T}$, and the target homogeneous network is defined as ${G}_{b}^{T}{G}_{b}$, which is a $n\times n$ matrix. In our task, taking the drug homogeneous matrix as an example (Fig. 2), the entry ${w}_{ij}^{d}$ with a higher value in this matrix can be interpreted as that the ${drug}_{i}$ and ${drug}_{j}$ would share more similar targets, and the similar principle can be applied to the target homogeneous matrix. Such a characteristic is in line with the known assumption – “guilt-by-association” [2]. Thus, the second type of homogeneous networks can carry more interactive information between drugs and between targets, which is helpful to improve the accuracy of the DTIs prediction.

Next, based on the corpora created by the truncated random walks, the Skip-gram model [28] is used to learn embeddings of the two types of vertices in the bipartite network (e.g., drug and target embeddings), which makes embeddings capture more high-order proximity information; Essentially, the purpose of the Skip-gram model is assigning the similar embeddings to the vertices which are more frequently co-occurred in the same context of a sequence in the corpora. Intuitively, if the vertices in a corpus sequence are more similar to each other, these vertices are more likely to co-occur in the same context, so they could be allocated more similar embeddings. Thus, we further add a relatively high restart probability (e.g., 0.7) to every step of truncated random walks. Taking the embedding process of drug nodes as an example, for a truncated random walk starting at a certain drug node, when the next node is randomly selected from the set which has other drug nodes having a connection to the current drug (the connection is determined based on the value between these two nodes in the drug homogeneous network, if the value is non-zero, which indicates that there is a connection between them), a number from 0 to 1 is randomly chosen. If this number is less than the restart probability, the next node will become the starting node instead. In this way, the drug nodes selected in the current corpus sequence are closer to the starting node, which could bring higher-quality embeddings for the DTIs prediction.

Therefore, in order to learn the implicit transition relationships, there needs two objection functions expressed in (2)—(3) to maximize the conditional probability for high-order proximities on the two corpora respectively, where $S$ denotes a vertex sequence which contains only ${u}_{i}$ nodes or only ${v}_{j}$ nodes, ${D}^{U}$ and ${D}^{V}$ correspond to the two generated corpora, ${C}_{S}({u}_{i})$ and ${C}_{S}({v}_{j})$ represent the context vertices of ${u}_{i}$ and ${v}_{j}$ in the sequence $S$, respectively, and context vertices are several vertices (the number is $\mathrm{ws}$ in total) before and after ${u}_{i}$ or ${v}_{j}$ in a sequence $S$. In addition, $P({u}_{c}|{u}_{i})$ refers to how likely ${u}_{c}$ is found in the contexts of ${u}_{i}$, and the similar meaning can be applied to $P({v}_{c}|{v}_{j})$.

2 $$\mathrm{maximize }{O}^{2}=\prod_{{u}_{i}\in S\wedge S\in {D}^{U}}\prod_{{u}_{c}\in {C}_{S}({u}_{i})}P({u}_{c}|{u}_{i})$$

3 $$\mathrm{maximize }{O}^{3}=\prod_{{v}_{j}\in S\wedge S\in {D}^{V}}\prod_{{v}_{c}\in {C}_{S}({v}_{j})}P({v}_{c}|{v}_{j})$$

Finally, the three parts of objection functions mentioned above can be integrated into a joint framework to capture explicit and implicit transition relationships simultaneously. The framework is optimized by the Stochastic Gradient Ascent (SGA) algorithm, which can be presented as the Eq. (4). $\mathrm{\alpha }$, $\beta$ and $\gamma$ are adjustable weights which control the relations between the three components.

4 $$\mathrm{maximize L}=\mathrm{\alpha log}{O}^{2}+\beta \mathrm{log}{O}^{3}-\gamma {O}^{1}$$

When optimizing the Eq. (4) using SGA, in order to save the calculation time, negative sampling [29], which approximates the costly denominator of the softmax function by sampling several negative instances, is adapted to learn the embedding vectors. As a result, the whole optimization process in one gradient step is as follows:

Firstly the $-\gamma {O}^{1}$ part is maximized to update embeddings $\overrightarrow{{u}_{i}}$ and $\overrightarrow{{v}_{j}}$ as the Eqs. (5)-(6):

5 $$\overrightarrow{{u}_{i}}=\overrightarrow{{u}_{i}}+\lambda \{\gamma {w}_{ij}[1-\sigma ({\overrightarrow{{u}_{i}}}^{T}\overrightarrow{{v}_{j}})]\bullet \overrightarrow{{v}_{j}}\}$$

6 $$\overrightarrow{{v}_{j}}=\overrightarrow{{v}_{j}}+\lambda \{\gamma {w}_{ij}[1-\sigma ({\overrightarrow{{u}_{i}}}^{T}\overrightarrow{{v}_{j}})]\bullet \overrightarrow{{u}_{i}}\}$$

where $\lambda$ is the learning rate and ${w}_{ij}$ is the weight of edge between ${u}_{i}$ and ${v}_{j}$ (in our study the weight is 1 if there is an edge between ${u}_{i}$ and ${v}_{j}$). Then, the $\mathrm{\alpha log}{O}^{2}$ and $\beta \mathrm{log}{O}^{3}$ parts are maximized separately for further updating the embedding vectors as follows:

7 $$\overrightarrow{{u}_{i}}=\overrightarrow{{u}_{i}}+\lambda \{\sum_{z\in \{{u}_{c}\}\cup {N}_{S}^{ns}({u}_{i})}\alpha [I\left(z,{u}_{i}\right)-\sigma ({\overrightarrow{{u}_{i}}}^{T}\overrightarrow{{\theta }_{z}})]\bullet \overrightarrow{{\theta }_{z}}\}$$

8 $$\overrightarrow{{v}_{j}}=\overrightarrow{{v}_{j}}+\lambda \{\sum_{z\in \{{v}_{c}\}\cup {N}_{S}^{ns}({v}_{j})}\beta [I\left(z,{v}_{j}\right)-\sigma ({\overrightarrow{{v}_{j}}}^{T}\overrightarrow{{\vartheta }_{z}})]\bullet \overrightarrow{{\vartheta }_{z}}\}$$

where ${u}_{c}$ and ${v}_{c}$ are the context vertices of ${u}_{i}$ and ${v}_{j}$ separately, ${N}_{S}^{ns}({u}_{i})$ denotes the negative samples (the number is $\mathrm{ns}$ in total) of ${u}_{i}$ in the sequence $S\epsilon {D}^{U}$, and the similar meaning can be applied to ${N}_{S}^{ns}({v}_{j})$. $I\left(z,{u}_{i}\right)$ and $I\left(z,{v}_{j}\right)$ are indicator functions determining whether vertex $z$ is the context vertex of ${u}_{i}$ and ${v}_{j}$ respectively (is: 1, not: 0). Besides, $\sigma$ is the sigmoid function $1/(1+{e}^{-x})$, and $\overrightarrow{{\theta }_{z}}$ and $\overrightarrow{{\vartheta }_{z}}$ are the embeddings of the context vertex of ${u}_{i}$ and ${v}_{j}$ respectively.

Furthermore, BiNE is an embedding method which could not well learn total isolation nodes that the truncated random walk cannot reach. However, under our transductive-learning setup, we reckon that the use of BiNE can be understood from another perspective. More specifically, many methods adopt multiple drug and target similarities (as a part of the input feature to generate homogeneous networks), which are pre-calculated over all nodes in the dataset based on certain properties of drugs and targets. As an analogy, we can treat BiNE as a similarity generator which takes drug and target Co-HITS matrices (that are calculated based on the whole bipartite DTI network) as the input to pre-calculate another type of similarity of drugs and targets. In this case, the form of this drug and target similarity is the embedding score, and the property on which it is based is the high-order proximity; and every node in the whole bipartite DTI network in used datasets has at least one edge such that the truncated random walk can produce every node’s (high-order proximity) similarity in advance (i.e., there are no isolation nodes actually in the process of high-order similarity production).

### Composite homogeneous network generation

As for the second step of our workflow, we choose a heuristic method to screen and combine different homogeneous networks (in matrix form) which contain different drug-drug and target-target similarity information [27]. This method can acquire an informative and robust composite homogeneous network by removing redundant information and integrating the retained features. Specifically, we first calculate the entropy of each homogeneous matrix for determining how much information these matrices contain. Secondly, delete the homogeneous matrices with the entropy value higher than $\mathrm{c}1\mathrm{log}\left(\mathrm{k}\right)$ where $\mathrm{c}1$ is a threshold to control the information each matrix contains (is subjectively set to 0.7) and $\mathrm{log}(\mathrm{k})$ represents the highest entropy contained among all matrices.

Next, flatten each matrix and calculate the Euclidean distance ($d$) between homogeneous matrices, and then start from the matrix with the lowest entropy, based on the similarity index ${E}_{s}$ (shown in Eq. (9)), further remove other matrices having ${E}_{s}$ higher than $\mathrm{c}2$ (is subjectively set to 0.6) with the current matrix, and the process will be repeated until all matrices are removed or retained. Finally, the similarity network fusion (SNF) [30] algorithm is adopted for non-linearly fusing the remaining matrices into a composite matrix that carries the necessary information from different similarity measures.

9 $${E}_{s}=\frac{1}{1+d}$$

As a result, a drug and a target composite matrix are obtained from multiple drug and target homogeneous matrices respectively. These two matrices and other matrices mentioned in this section all belong to the first type of the homogeneous network mentioned in the “Learning bipartite DTI embedding” section, which sizes are $m\times m$ (for drug) and $n\times n$ (for target), respectively.

### Generating new embedding vectors of drug-target pairs

**Fig. 3:**
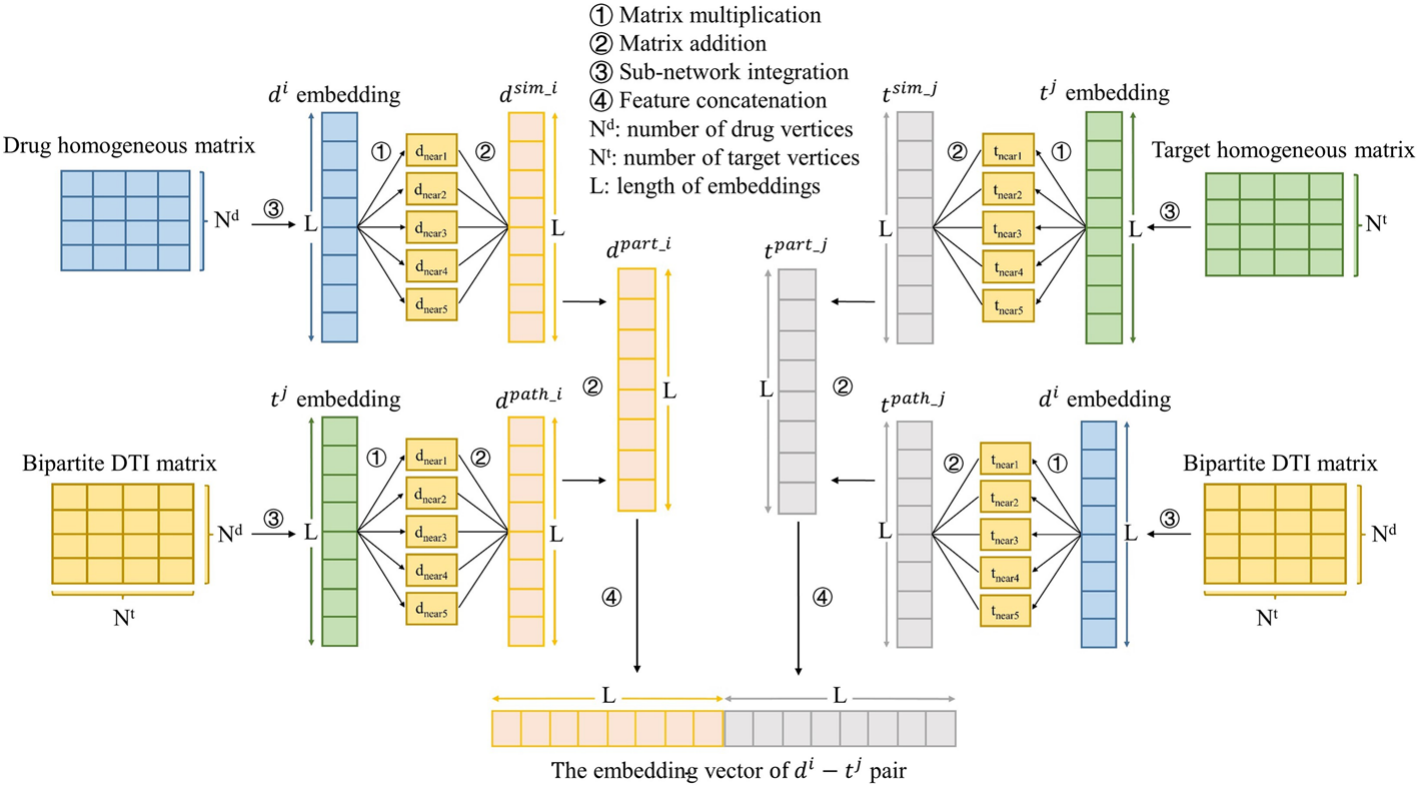
The illustration of the embedding generation process of the ${d}^{i}-{t}^{j}$ pair. Characteristics from three types of sub-networks will be combined to create a new embedding representation. This process will be repeated many times until embeddings of all drug-target pairs in the DTIs space are produced

In order to tackle the problem that some recent embedding-based methods cannot add the pathway information about drug-target interactions into embeddings of drug-target pairs (e.g., simply concatenating generated drug and target embeddings as the final embeddings of drug-target pairs), we provide a method, which draws on the path-based information (about similar drugs interacting with the same targets and about similar targets sharing the same drugs), to acquire new embeddings of every drug-target pair (i.e., the reconstruction of DTI relations (network) included in the whole dataset). The intuition behind this idea is that, although separate drug and target embeddings produced by embedding algorithms could carry certain DTI (high-order proximity) information through learning process, the characterization of DTIs they contain for DTI predictions is still insufficient before the heterogeneous information (e.g., path-based knowledge) is added. The explanation of main calculation steps is shown in Fig. 3.

Specifically, taking the embedding generation process of a ${d}^{i}-{t}^{j}$ pair as an example, first, we obtain ${d}^{i}$ and ${t}^{j}$ embeddings ($\overrightarrow{{d}^{i}}$ and $\overrightarrow{{t}^{j}}$) produced by BiNE, the bipartite DTI matrix ${G}_{b}$, and drug and target homogeneous fused matrices mentioned in the “Composite similarity matrix generation” section. Second, acquire the five nearest drugs of ${d}^{i}$ according to the weights in the drug homogeneous matrix. That is, find the row corresponding to ${d}^{i}$ in the drug homogeneous matrix, and the values in the row are sorted from large to small, then the drugs corresponding to the five largest values are selected. In the same way, five targets with the highest similarity to ${t}^{j}$ can be found.

Third, multiply the embedding vector of ${d}^{i}$ by corresponding weights (i.e., similarities) of selected five nearest drugs in the drug homogeneous matrix respectively, then sum the obtained five products up to acquire a new feature ${d}^{sim\_i}$; the same rule can be applied to the embedding vector of ${t}^{j}$ to acquire a new feature ${t}^{sim\_j}$ (Eqs. (10)-(11)).

10 $${d}^{sim\_i}=\sum_{{d}^{z}\in {D}^{near}}{w}_{d}^{z}\overrightarrow{{d}^{i}}$$

11 $${t}^{sim\_j}=\sum_{{t}^{z}\in {T}^{near}}{w}_{t}^{z}\overrightarrow{{t}^{j}}$$

where ${D}^{near}$ and ${T}^{near}$ denote the set of the selected nearest drugs of ${d}^{i}$ and the nearest targets of ${t}^{j}$ separately, ${w}_{d}^{z}$ is the weight between ${d}^{z}$ and ${d}^{i}$ in the drug homogeneous matrix, and the similar meaning can be applied to ${w}_{t}^{z}$. The main purpose in this step is integrating drug-drug and target-target homogeneous matrices (similarity information) into the embedding vectors ${d}^{i}$ and ${t}^{j}$, respectively. In the fourth step, multiply the embedding vector ${t}^{j}$ by weights in ${G}_{b}$ between selected five nearest drugs and ${t}^{j}$ respectively, and then sum the five generated products up for acquiring a new feature ${d}^{path\_i}$. At the same time, we multiply the embedding vector ${d}^{i}$ by weights in ${G}_{b}$ between five selected nearest targets and ${d}^{i}$ respectively, and then sum the obtained products up to create a new feature ${t}^{path\_j}$ (Eqs. (12)-(13)).

12 $${d}^{path\_i}=\sum_{{d}^{z}\in {D}^{near}}{w}_{{t}^{j}}^{z}\overrightarrow{{t}^{j}}$$

13 $${t}^{path\_j}=\sum_{{t}^{z}\in {T}^{near}}{w}_{{d}^{i}}^{z}\overrightarrow{{d}^{i}}$$

where ${w}_{{t}^{j}}^{z}$ and ${w}_{{d}^{i}}^{z}$ represent the weight between ${d}^{z}$ and ${t}^{j}$ in ${G}_{b}$ and the weight between ${t}^{z}$ and ${d}^{i}$ in ${G}_{b}$, respectively. In this step, we can model the interactive pathway information about the known interactions between drugs (which are more similar to ${d}^{i}$) and ${t}^{j}$ as well as the known interactions between ${d}^{i}$ and targets (which are more similar to ${t}^{j}$). In the fifth step, a new embedding vector ${d}^{part\_i}$ is calculated by summing the vectors ${d}^{sim\_i}$ and ${d}^{path\_i}$ up, and the embedding vector ${t}^{part\_j}$ is formed in a similar way (Eqs. (14)-(15)).

14 $${d}^{part\_i}={d}^{sim\_i}+{d}^{path\_i}$$

15 $${t}^{part\_j}={t}^{sim\_j}+{t}^{path\_j}$$

Finally, the ${d}^{part\_i}$ and ${t}^{part\_j}$ can be concatenated to obtain an embedding of the ${d}^{i}-{t}^{j}$ pair, which effectively integrates characteristics from the bipartite DTI network as well as drug and target homogeneous networks. In addition, this calculation process is conducted after the cross-validation (CV) setup.

### RF-based drug-target interaction predictor

After acquiring embeddings of all drug-target pairs in the dataset, the RF classifier [31] can be used for predicting the DTIs. RF has been proved to perform well in the face of high-dimensional features and be able to deal with overfitting in the case of insufficient training data. More importantly, it can handle the sample-class-imbalance problem efficiently. We implement the RF classifier by using the scikit-learn [32] tool, and the embeddings of drug-target pairs are as the input. The probability of whether each drug-target pair has a potential interaction is then predicted.

In addition, we tune the parameters of the RF classifier for better learning the complex integrated embeddings. The number of estimators is set to 100, the criterion for measuring the quality of a split is the Gini coefficient, and we make the weights of the model inversely proportional to the occurrence frequency of positive (known DTIs) and negative (unknown DTIs) classes based on input labels, to further overcome the challenge of considerable imbalance between the number of known and unknown DTIs.

## Results

In this section, we evaluate the predictive performance of the purposed method in two different settings (S^D^, S^T^) based on two main datasets. Firstly, we introduce model parameters, details of experimental settings as well as model evaluation metrics. Then, we compare our method with other advanced DTI prediction approaches under the same experimental conditions. Next, we conduct a case study in which unknown DTIs are predicted and top-score results are validated by searching for the evidence from multiple reference databases.

### Dataset

In this study, two benchmark datasets are used for establishing the bipartite DTI relations (networks); the first one (a gold standard dataset) was collected by Yamanishi et al. [33], which includes four DTI subsets classified by the types of target proteins (in human): Enzymes (E, including 445 drugs and 664 proteins), Ion Channels (IC, 210 drugs and 204 proteins), G-protein-coupled Receptors (GPCR, 223 drugs and 95 proteins), and Nuclear Receptors (NR, 54 drugs and 26 targets), respectively. The second one was obtained from Olayan RS et al. [27], consisting of interactions between 1482 FDA-approved drugs and 1408 human target proteins (including multiple categories), which were acquired from the DrugBank dataset [34]. Furthermore, the proportion of known and unknown interactions in these datasets are shown in Table 1.

**Table 1:** The proportion of positive and negative samples in each dataset

	Nuclear receptors	Ion channels	GPCR	Enzymes	DrugBank
Positive	6.41% (90)	3.45% (1476)	3% (635)	1% (2926)	0.47% (9881)
Negative	93.59% (1314)	96.55% (41,364)	97% (20,550)	99% (292,554)	99.53% (2,076,775)

In the bipartite DTI networks, if there is a known interaction between ${d}^{i}$ and ${t}^{j}$, the corresponding weight is 1, otherwise it is 0 instead.

Besides, the drug-drug and target-target similarities for generating the composite homogeneous network were obtained from Olayan RS et al. [27]. As for the similarities for the first dataset, there are three types of drug similarities (chemical structure fingerprints, drug side-effects profiles, and the Gaussian interaction profile (GIP)) and six varieties for targets (amino acid sequences profiles, various parameterizations of the Mismatch, Spectrum kernels, target proteins functional annotation based on Gene Ontology (GO) terms, proximity in the protein–protein interaction (PPI) network, and the GIP). With regards to the second dataset, there are eight similarities for drugs (molecular fingerprints, drug interaction profiles, side-effects profiles, drug profiles of the anatomical therapeutic class coding system, drug-induced gene expression profiles, drug disease profiles, drug pathways profiles, and the GIP), and six in total for targets (protein amino acid sequence, protein GO annotations, proximity in the PPI network, the GIP, protein domain profiles, and gene expression similarity profiles of protein encoding genes). Besides, the weights in each kind of similarity matrix were mapped to the same scale using the 0-1 normalization method.

### Experimental settings, evaluation metrics and model parameters

In order to avoid an overly idealistic assessment, we evaluate the performance of our method (i.e., the quality of generated embeddings) under two different DTI prediction settings inspired by Pahikkala T et al. [35], which provide different split of generated drug-target pair embeddings set. Further, same to the definition of the settings in Olayan RS et al. [27], the first setting is called the S^D^ task in which the tenfold CV is used, and in each fold, drug-target pair embeddings in the DTIs space corresponding to one tenth of all drugs will only appear in the test set). As an analogy, for the S^T^ task, drug-target pairs in the DTIs space corresponding to one tenth of all targets will only appear in the test set. In addition, the case study mentioned above corresponds to a more realistic scenario to test the performance of predicting unknown DTIs, in which all known DTIs are added to the training data as the auxiliary information to predict unknown DTIs (and then verify these predictions) [27, 36]. More specifically, we first set the labels of all known DTIs to 1, and the labels of other samples (including drug-target pairs without any interaction and drug-target pairs with undiscovered interactions) in the DTIs space are set to 0. Then, we randomly divide all drug-target pairs labeled 0 into 10 non-overlapping groups, and in each group, all samples labeled 1 are incorporated into the training set. Thus, during the whole predictive process, the RF classifier will receive embeddings corresponding to all drug-target pairs labeled 0 and thus can provide the probability scores of all unknown drug-target pairs in the given dataset, so that we can acquire predicted novel DTIs from top-ranked-score results. Furthermore, since the aim of the case study is to predict potential interactions of unknown DTIs only, it is not necessary to calculate the performance metrics.

As for the S^D^ and S^T^ tasks, we can acquire a more reasonable performance estimation by choosing the PR-AUC as the main evaluation metrics, it functions well when there are far more negative samples than positive samples in the dataset (Table 1), because it can impose a stricter punishment on the false positive (FP) case [37], and the ROC-AUC is selected as the auxiliary evaluation metrics. In each fold of CV, the PR-AUC is obtained by calculating the area under the precision-recall (PR) curve constructed based on the predictions of the RF classifier and corresponding actual labels. Similarly, the ROC-AUC can be calculated from the ROC curve, which is plotted by multiple true positive rate (TPR)—false positive rate (FPR) pairs under different threshold settings. The overall PR-AUC and ROC-AUC of the tenfold CV are derived by averaging the values in all folds. The general hyperparameters of our method tuned by the grid search for each dataset are shown in Table 2. In addition, the dimension of final embeddings of drug-target pairs is twice as high as that of the embeddings generated by BiNE.

**Table 2:** Hyperparameters of BiNE for different datasets

Parameter names	Nuclear receptors	Ion channels	GPCR	Enzymes	DrugBank
Maximum iterations	100	100	100	1000	100
Learning rate	0.1	0.1	0.1	0.1	0.01
Number of negative samples $\mathrm{ns}$	4	4	4	4	2
Size of window $\mathrm{ws}$	5	7	5	5	5
Trade-off parameter α	0.01	0.01	0.01	0.01	0.01
Trade-off parameter β	0.1	0.1	0.1	0.1	0.01
Trade-off parameter γ	0.1	1	0.1	0.1	0.1
Embedding size of BiNE	64	128	32	128	128
Embedding size of drug-target pairs	128	256	64	256	256
Walk stopping probability	0.15	0.15	0.15	0.15	0.15
Walk restart probability	0.7	0.7	0	0.7	0.7

### Comparison with other recent DTI prediction methods

In this section, under the same datasets, evaluation metrics, and prediction tasks (S^D^ and S^T^ tasks), seven advanced methods including DDR [27], NEDD [38], NRLMFβ [39], DTINet [2], CMF [40], BLM-NII [41], and NetLapRLS [10] which can effectively utilize the drug-target related knowledge are involved into the performance comparison, which allows us to compare the proposed method with the representative heterogeneous-network-based, matrix-factorization-based, and kernel-based methods. For methods which could only handle a single type of drug and target similarities, like BLM-NII and NetLapRLS, we use compound structure similarities (for drugs) and protein sequence similarities (for targets) provided by Yamanishi et al. [33] as the model input. In order to further demonstrate the effectiveness and feasibility of integrating similarity-based and path-based prior knowledge into the embeddings of drug-target pairs, we add BiNE into the comparison. That is, obtain the embedding vector of each drug-target pair by directly concatenating corresponding drug and target embeddings produced by BiNE (i.e., not considering any additional prior information). The generated vectors are then put into a RF classifier which is the same as the RF used in our method, to get the probability score of every drug-target pair. In addition, we do not consider DTiGEMS + [16] mentioned above, because it is difficult to evaluate this method and ours simultaneously in the same experimental settings. In other words, it requires the same number of positive and negative samples in each fold of a tenfold CV, while in our method, the allocation of samples follows the rule of the S^D^ and S^T^ tasks, which results in highly imbalanced samples in the training set.

Tables 3 and 4 show the PR-AUC and ROC-AUC of the methods participating in the S^D^ and S^T^ tasks. In general, based on the main evaluation metrics PR-AUC, our method has overall better performance than the other methods in the both tasks. For the S^D^ task, the PR-AUC achieved by our method increases by 1.2%, 2.6%, 3.2%, 2.8%, and 35.1% on E, IC, GPCR, NR, and DrugBank datasets, respectively, compared with that of the second-best model. For the S^T^ task, the corresponding improvements made by our method are 1.8% (E), 2.8% (IC), 4.2% (GPCR), 13.8% (NR), and − 11.7% (DrugBank), respectively. Meanwhile, under the auxiliary evaluation metrics ROC-AUC, our method is also generally superior to other models.

**Table 3:** Performance comparison over five datasets in the S^D^ task

Methods	Performance indicators	Datasets				
		E	IC	GPCR	NR	DrugBank
DTI-HeNE	PR-AUC	**0.908**	**0.912**	**0.982**	**0.980**	**0.921**
	ROC-AUC	0.980	0.981	**0.998**	**0.997**	**0.995**
NEDD	PR-AUC	0.896	0.879	0.951	0.953	0.578
	ROC-AUC	**0.986**	0.982	**0.998**	0.993	0.959
DDR	PR-AUC	0.897	0.888	0.857	0.874	0.598
	ROC-AUC	0.972	**0.983**	0.958	0.941	0.895
NRLMFβ	PR-AUC	0.313	0.322	0.324	0.471	0.249
	ROC-AUC	0.679	0.735	0.824	0.892	0.669
DTINet	PR-AUC	0.270	0.320	0.298	0.250	0.316
	ROC-AUC	0.772	0.727	0.764	0.685	0.896
CMF	PR-AUC	0.193	0.190	0.355	0.450	0.047
	ROC-AUC	0.793	0.701	0.854	0.817	0.600
BLM-NII	PR-AUC	0.076	0.209	0.099	0.417	0.185
	ROC-AUC	0.752	0.754	0.593	0.792	0.884
NetLapRLS	PR-AUC	0.112	0.178	0.223	0.416	0.120
	ROC-AUC	0.782	0.750	0.806	0.782	0.855
BiNE	PR-AUC	0.766	0.751	0.588	0.724	0.316
	ROC-AUC	0.944	0.962	0.944	0.936	0.856

Best performing methods under the current dataset and performance indicator are indicated in bold

**Table 4:** Performance comparison over five datasets in the S^T^ task

Methods	Performance indicators	Datasets				
		E	IC	GPCR	NR	DrugBank
DTI-HeNE	PR-AUC	**0.941**	**0.974**	**0.914**	**0.989**	0.429
	ROC-AUC	**0.997**	**0.997**	0.976	**0.996**	**0.891**
NEDD	PR-AUC	0.929	0.901	0.876	0.853	0.421
	ROC-AUC	0.992	0.982	**0.995**	0.983	0.881
DDR	PR-AUC	0.924	0.947	0.862	0.818	**0.486**
	ROC-AUC	0.974	0.987	0.964	0.929	0.885
NRLMFβ	PR-AUC	0.797	0.791	0.527	0.541	0.268
	ROC-AUC	0.931	0.954	0.939	0.921	0.766
DTINet	PR-AUC	0.477	0.425	0.093	0.272	0.176
	ROC-AUC	0.895	0.860	0.681	0.676	0.841
CMF	PR-AUC	0.273	0.365	0.402	0.366	0.104
	ROC-AUC	0.765	0.754	0.809	0.533	0.702
BLM-NII	PR-AUC	0.650	0.738	0.352	0.418	0.158
	ROC-AUC	0.911	0.914	0.775	0.533	0.831
NetLapRLS	PR-AUC	0.651	0.708	0.302	0.348	0.140
	ROC-AUC	0.907	0.912	0.758	0.523	0.810
BiNE	PR-AUC	0.674	0.612	0.432	0.459	0.183
	ROC-AUC	0.936	0.919	0.831	0.651	0.841

Best performing methods under the current dataset and performance indicator are indicated in bold

To investigate why our method performed differently in the S^D^ and S^T^ tasks on the DrugBank dataset, we counted the number of targets that every drug had (${N}^{drug}$) in the S^D^ task (in which data was split according to drugs) and the number of drugs that every target corresponded to (${N}^{target}$) in the S^T^ task (in which data was split according to targets) based on the known DTIs in the DrugBank dataset; and we further calculated the mean and variance of ${N}^{drug}$ and ${N}^{target}$. The corresponding values were $\mathrm{Mean}\left({N}^{drug}\right)=6.67$, $\mathrm{Var}\left({N}^{drug}\right)=45.30$, $\mathrm{Mean}\left({N}^{target}\right)=7.02$, and $\mathrm{Var}\left({N}^{target}\right)=660.80$, respectively. The significant difference between $\mathrm{Var}\left({N}^{drug}\right)$ and $\mathrm{Var}\left({N}^{target}\right)$ components indicates that when auxiliary information (i.e., pathway and similarity-based information), $\mathrm{Mean}\left({N}^{drug}\right)$, and $\mathrm{Mean}\left({N}^{target}\right)$ are similar, because our method is dependent on high-quality bipartite DTI relations to produce embeddings as well as the sample variance related to DTI relations in the S^T^ task is much larger than that in the S^D^ task, therefore, our method performs better in the S^D^ task than in the S^T^ task. Meanwhile, another heterogeneous network embedding method DTINet, which also relies on DTIs to generate projection matrix for DTI predictions, also suffers a significant drop in the predictive performance (from 0.316 to 0.176 in PR-AUC). In contrast, for DDR, since it is not an embedding-based method that needs DTIs, therefore, its performance in the S^T^ task remains stable. This phenomenon can also prove that the quality of bipartite DTI relations plays a significant role for learning embeddings of a heterogeneous DTI network.

In addition, after obtaining drug and target embeddings, DTINet used inductive matrix completion (IMC) to learn these embeddings and known DTIs directly, for generating a projection matrix, which led to DTI predictions, and there were few between-class imbalance learning techniques being adopted. While our method utilized the RF classifier to predict DTIs, which could handle the sample-class-imbalance problem more efficiently. Therefore, in the face of highly imbalanced samples in the S^D^ and S^T^ tasks, our method outperformed DTINet.

### Case study

To further prove the capability of the proposed model in a more realistic DTI prediction scenario, we introduce the case study mentioned in the “Experimental settings, evaluation metrics and other model parameters” section. Based on the case study, we can acquire drug-target pairs with the highest (top 5) probability scores predicted by the RF classifier on each dataset and search for the relevant evidence from six external databases (KEGG (K) [42], DrugBank (D) [34], Matador (M) [43], ChEMBL (C) [44], T3DB (T) [45], and CTD [46]). The DTIs contained in the used datasets were collected before 2008, thus, we can do verification by using newly updated DTIs in the above databases. The predicted interactions (a total of 25 pieces of data) and corresponding supporting evidence are shown in Table 5.

**Table 5:** The novel interactions predicted by DTI-HeNE and corresponding evidence provided by external databases

Drug ID	Drug names	Target ID	Target names	Evidence sources
*Enzymes*				
D00437	Nifedipine	hsa1585	CYP11B2	M, CTD
D00528	Caffeine	hsa50940	PDE11A	CTD
D00126	Ibuprofen	hsa247	ALOX15B	M
D00394	Trimipramine	hsa5152	PDE9A	None
D00574	Aminoglutethimide	hsa1589	CYP21A2	M
*G-protein-coupled receptors*				
D00513	Pindolol	hsa152	ADRA2C	None
D01713	Epinastine	hsa152	ADRA2C	C
D00454	Olanzapine	hsa3357	HTR2B	K, T, CTD
D02354	Thiethylperazine	hsa1816	DRD5	C
D00283	Clozapine	hsa1132	CHRM4	D, C, T
*Ion channels*				
D00524	Carbachol	hsa1138	CHRNA5	CTD
D03365	Nicotine	hsa1138	CHRNA5	K, D, T, CTD
D03826	Physostigmine sulfate	hsa2564	GABRE	None
D00550	Midazolam	hsa2570	GABRR2	T, CTD
D00303	Disopyramide	hsa6326	SCN2A	K
*Nuclear receptors*				
D00585	Mifepristone	hsa2099	ESR1	M, T, CTD
D00066	Progesterone	hsa2100	ESR2	D, CTD
D01161	Fulvestrant	hsa5241	PGR	M, C
D00182	Norethisterone	hsa2099	ESR1	CTD
D00066	Progesterone	hsa367	AR	DB, CTD
*DrugBank*				
DB01589	Quazepam	P47870	GABRB2	T, K
DB00546	Adinazolam	Q16445	GABRA6	K
DB00321	Amitriptyline	P14416	DRD2	T
DB01215	Estazolam	Q16445	GABRA6	K
DB00696	Ergotamine	P41595	HTR2B	D, C, T

In summary, we found the evidence for the majority of predicted interactions (22 out of 25), and we carried out further research on these predictions. For the drug in the drug-target pair having a top probability score, we can usually find the evidence that this drug can interact with other targets which belong to the same gene family as the target in this drug-target pair. For example, in the GPCR group, the first ranked prediction indicates that there is a potential interaction between pindolol and ADRA2C. Pindolol is a moderately lipophilic beta blocker (adrenergic beta-antagonists) [47], and ADRA2C stands for the Alpha-2C adrenergic receptor. It was reported that the gene coding ADRA2C is associated with beta blockers response in a group of patients troubled by chronic kidney disease [48]. Meanwhile, we find that ADRA2A and ADRA2B, which are also members of the ADRA gene family, can interact with pindolol (from the Matador database).

There is another instance that can be used to further illustrate such a characteristic of DTI predictions. In the IC group, it was predicted that carbachol could react with CHRNA5 (the top ranked interaction). Carbachol [49] is a slowly hydrolyzed cholinergic agonist and CHRNA5 refers to the neuronal acetylcholine receptor subunit alpha-5. There is a recent drug-repurposing report that carbachol can combine with histamine and dopamine to block the inhibitory effects of benztropine mesylate on mammosphere formation of breast cancer stem cells. During the interaction process, the mRNA expression levels of CHRNA5 were variably altered within different types of tested cells [50]. Furthermore, the interactive information between carbachol and CHRNA2, CHRNA3, CHRNA4, CHRNA6 can be accessed from the Matador dataset.

## Discussion

In this work, we introduce a novel DTI prediction method – DTI-HeNE, which resorts to the heterogeneous information from every sub-network of the heterogeneous DTI network, to produce high-quality embeddings of drug-target pairs. Under the same experimental settings (S^D^ and S^T^ tasks) and evaluation metrics (PC-AUC, ROC-AUC), we obtained the comparison results shown in Tables 3 and 4. Based on current five benchmark datasets, we show that the overall performance of our method is better than that of other advanced methods involved in the experiment. We consider that the superior performance of DTI-HeNE is attributed to the following two reasons.

The first reason is the use of BiNE, when processing bipartite DTI relations for DTI predictions, in addition to modeling observed edges between drugs and targets, it is essential to consider the distinctive information of drug and target nodes, respectively. BiNE implements this by separately extracting implicit transition relationships between drugs and between targets (i.e., acquiring the 2nd-order proximity between the same type of vertices), which can provide unique similarity information (e.g., the homogeneous network illustrated in Fig. 2) compared with the similarities calculated based on domain knowledge. The second reason is that distinct information of each sub-network of the heterogeneous DTI network is effectively combined by using the path-based semantic information, as integrating this information through interpretable pathways between the sub-networks could contribute to a more explicit description of drug-target associations throughout the DTIs space. For the analogical reason, DDR also achieved great performance by extracting various path-category-based features from a heterogeneous network and combining the generated features into one fixed-length vector (as a representation of one drug-target pair). The advantage of our method is that the high-order prior proximity information of drugs and targets can be fused into the representations of drug-target pairs, and the length of these representations is no longer fixed so that we can flexibly adjust the length to meet the needs of some specific tasks. These benefits are brought by utilizing embedding-based algorithm as the backbone to process the heterogeneous DTI network.

When doing the case study, we observed that, for the newly discovered DTIs, it was common to find the supporting evidence that the targets that belonged to the same gene family as the predicted target could interact with the predicted drug. We speculate the reason is that we follow the principle “similar drugs may interact with similar targets” to design the predictive method, which can be reflected in the process of the Co-HITS-based homogeneous matrix generation and the drug-target embedding generation. The benefit is that we can forecast unknown DTIs more purposefully and directionally and reduce the probability of misjudgment using abundant similarity information. However, the scale of the searching space in which novel DTIs could be found is also narrowed. That is, if the similarity between the nodes in a certain drug-target pair and other nodes in the dataset is relatively low, it is less likely for this drug-target pair to be predicted to have a potential interaction, even though it actually contains an association. Thus, we plan to explore how to give our method a functional extension which can give higher attention to certain drugs having relatively lower similarity to other drugs but are worthy of further study. In addition, the proposed method is an attempt to use the stage-by-stage transductive-learning method to do the DTIs prediction, the benefit is that the method has better interpretability than many end-to-end methods, as every stage has a clearly actual meaning in the workflow; however, it is because currently our method functions in a transductive-like way, it has higher computational cost than the inductive-learning method (as the inductive learning will not be limited to a specific dataset, e.g., fixed drugs and targets, i.e., transductive learning can bring higher predictive accuracy than inductive learning due to the better use of additional information of unknown samples in the dataset with sparse known interactions, while the model have to be re-run if any new nodes/samples will be added into the dataset). Thus, in the future, we would like to do further modification of our method to make it suitable for inductive-like DTI prediction task.

Furthermore, adapting our algorithm to predict interactions between microRNAs (miRNAs) and small molecular drugs would be a highly interesting future direction. This is because increasing number of studies have found that the abnormal expression of miRNAs had close connections with many complex human diseases, and small molecular drugs could treat them by modulating the expression of miRNAs [51]. Similar to the general drug-target interaction prediction, accurate predictions of miRNA targets of small molecular drugs can be made based on miRNA and small molecule similarity networks, known miRNA-molecule interactions, and the “guilt-by-association” assumption [52–54]; and such data is quite similar to the required data of our method. Based on this, we believe that, with proper adjustments and data, DTI-HeNE can be applied to predict the interaction between small molecular drugs and miRNAs.

## Conclusion

In this paper, a novel heterogeneous network embedding method – DTI-HeNE, has been proposed for the DTI prediction, which can extract distinct features from every sub-network of the heterogeneous DTI network and concatenate these features by the topological information between the sub-networks. This study has demonstrated the feasibility and practicability of de-constructing the heterogeneous DTI network to capture the contained complex information for generating high-quality embeddings of drug-target pairs. In addition, we have proved that, after proper adjustments, BiNE can efficiently learn the special bipartite relations included in the drug-target interactions.

Moreover, our method achieved overall higher predictive accuracy than other advanced methods in different experimental scenarios based on the same way of evaluation and verification. In the task of novel DTI predictions, our method can also generate reasonable results with clear directivity. In conclusion, for drug repurposing, the proposed method is an effective and useful tool to identify new DTIs.

## Data Availability

The datasets analyzed during the current study are available in the DDR repository, https://bitbucket.org/RSO24/ddr/. The source codes are publicly available in the GitHub repository, https://github.com/arantir123/DTI-hene/.

## Abbreviations

DTI: Drug-target interaction
BiNE: Bipartite network embedding
RF: Random forest
FDA: Food and Drug Administration
3D: Three-dimensional
RWR: Random walk with restart
DAE: Denoising autoencoder
CNN: Convolutional neural network
GCN: Graph convolutional network
SGA: Stochastic gradient ascent
SNF: Similarity network fusion
IMC: Inductive matrix completion
E: Enzymes
IC: Ion channels
GPCR: G-protein-coupled receptors
NR: Nuclear receptors
GIP: Gaussian interaction profile
GO: Gene ontology
PPI: Protein–protein interaction
CV: Cross-validation
FP: False positive
PR: Precision-recall
TPR: True positive rate
FPR: False positive rate
K: KEGG
D: DrugBank
M: Matador
C: ChEMBL
T: T3DB
MicroRNA: MiRNA
